# Nano-chitosan-coated, green-synthesized selenium nanoparticles as a novel antifungal agent against *Sclerotinia sclerotiorum*: in vitro study

**DOI:** 10.1038/s41598-024-79574-x

**Published:** 2025-01-06

**Authors:** Mohamed M. Desouky, Radwa H. Abou-Saleh, Tarek A. A. Moussa, Heba M. Fahmy

**Affiliations:** 1 Biophysics Department, Faculty of Science, Cairo University, Giza, Egypt; 2 Nanoscience and Technology Program, Faculty of Science, Galala University, Galala City, New Galala City, 43511 Suez Egypt; 3 Biophysics Group, Physics Department, Faculty of Science, Mansoura University, Mansoura, Egypt; 4 Botany and Microbiology Department, Faculty of Science, Cairo University, Giza, Egypt

**Keywords:** Green Synthesis, Chitosan, Selenium, Nanocomposite, *Sclerotinia Sclerotiorum*, Biotechnology, Microbiology

## Abstract

Chemical fungicides have been used to control fungal diseases like *Sclerotinia sclerotiorum*. These fungicides must be restricted because of their toxicity and the development of resistance strains. Therefore, utilizing natural nanoscale materials in agricultural production is a potential alternative. This work aimed to investigate the antifungal properties of a nanocomposite (nano-chitosan-coated, green-synthesized selenium nanoparticles) against the plant pathogenic fungus *S. sclerotiorum*. Chemical reduction was used to produce selenium nanoparticles from citrus peel extracts, and ionotropic gelation was used to produce chitosan nanoparticles. The nanocomposite has been produced using selenium nanoparticles stabilized by chitosan and cross-linked with sodium tripolyphosphate. Transmission electron microscopy, dynamic light scattering, X-ray diffraction, UV-VIS spectroscopy, and Fourier transform infrared spectroscopy were used to characterize all produced nanostructures. The in vitro antifungal activity and minimum inhibitory concentration of all bulk and nanostructures are investigated at (0.5, 1, 5, 10, 50, 100) ppm concentrations. Scanning electron microscopy was used to detect structural deformations in the fungal mycelium. The findings support the successful synthesis and characterization of all nanoparticles. Lemon peel extract produced smaller, more stable, and distributed selenium nanoparticles (42.28 ± 18.5 nm) than orange peel extract (85.7 ± 140.22 nm). Nanostructures, particularly nanocomposite, have shown a considerable increase in antifungal efficacy compared to bulk structures. At a minimum inhibitory concentration of 0.5 ppm, the nanocomposite exhibited 100% inhibitory activity. The nanocomposite with a concentration of 0.5 ppm exhibited the lowest average fungal biomass (0.32 ± 0.05 g) among all tested nanostructures. Fungal hyphae treated with 0.5 ppm of nanocomposite within 18 h of treatment revealed substantial damage and deformation. These results provide new insights into the nanocomposite as an eco-friendly and promising antifungal agent against other plant pathogenic fungi.

## Introduction

*S. sclerotiorum*, or white mould, generally has different adverse effects on plant development. For instance, it can cause decreases in the weight of the plant’s stem and root, whether fresh or dry, resulting in the death of host tissues^1^. Oxalic acid and cell wall-degrading enzymes (CWDEs) are considered early toxins of *S. sclerotiorum* pathogenicity, followed by nutrient elimination from the host cell^2^. Despite being a necrotrophic pathogen, *it* enters a brief biotrophic phase, which lasts for half or one day after infection^3^. It inhibits the host’s defensive barriers during the phase^4^. Following this, the hyphae of subcutaneous *S. sclerotiorum* extend into many layers of cells. Upon successfully colonizing its branching hyphae, *S. sclerotiorum* transitions into a necrotrophic stage, producing substantial quantities of reactive oxygen species, toxins, and cell wall-degrading enzymes (CWDEs). This process ultimately results in the induction of host cell death and the manifestation of necrotic symptoms^5–7^.

Recent studies demonstrate the antifungal action of selenium against *S. sclerotiorum* as a natural material in the form of sodium selenite (SS)^8,9^. Selenium can protect human and animal body tissues from damage caused by free radicals^10^. However, selenate and selenite forms of selenium have the highest toxic effects because of their high solubility and bioavailability^10^. In contrast, Nano-selenium has an advantage over bulk selenium because it can be employed in its zero-oxidation state (Se^0^), which has low toxicity and significant bioavailability Compared to the bulk state^11,12^. Furthermore, Selenium nanoparticles (Se NPs) have gained interest for usage in agriculture during the last ten years owing to their increased antioxidant and antimicrobial effects with low toxicity compared to bulky selenium^13^.

The antifungal activity of the chemically synthesized Se NPs against *S. sclerotiorum* was recently tested^14^, offering a great chance to fight *S. sclerotiorum*. However, producing Se NPs via chemical or physical synthesis has many limitations, including several steps during the synthesis process, significant energy consumption, expensive costs, and toxic effects^15^. Biogenic synthesis, on the other hand, is a viable, low-cost, and echo-friendly alternative because it utilizes natural resources such as microbes and plant materials^16–18^. Using plant material extracts to reduce metal ions into nanoparticles is considered a single-step green synthesis process^19^. Because of their notable activities, citrus extracts have been widely employed for synthesizing metallic nanoparticles^17,18^. Ascorbic acid, three times more abundant in citrus peels than other fruit parts, is a powerful reducing agent that can effectively mitigate SS to Se NPs^15,20^. Therefore, citrus reticulate peel extracts as plant material have been selected to be highly effective in reducing SS into spherical Se NPs compared to other plant extracts^15,21^.

Chitosan(CS) is a potential natural fungicide selected in this study with Se NPs to treat postharvest infections in fruits and vegetables^22,23^. The results showed that the treatment with CS at its optimum inhibitory concentration of 0.2% (w/v) caused host resistance to *S. sclerotiorum*^24^. Other studies looked at the antifungal activity of CS and chitosan nanoparticles (CS NPs) at the optimal inhibitory concentrations of 1% (w/v) against *S. sclerotiorum*, causing extensive damage to the plasma membrane^23,25^.

The current study details a systematic approach for preparing and characterizing nano-chitosan-coated green synthesized selenium nanoparticles (NCS-Se NPs). CS NPs will play a dual role as a stabilizing agent for Se NPs and as a natural antifungal agent with Se NPs against the same causal pathogen, *S. sclerotiorum*. In vitro studies tested the antifungal activity of different concentrations of the bulk and Nanoparticles, compared to the whole nanocomposite (NCS-Se NPs), to study its synergistic effect and determine the minimum inhibitory concentration (MIC). The antifungal activity of the nanostructures was tested at the same concentrations of their bulk forms, influenced by their particle size. The size is often one of the most critical factors affecting the antifungal activity of the tested material^26–28^. The morphological changes of the pathogen after treatment were also detected.

## Results and discussion

### Identification of the fungal isolate

**Fig. 1:**
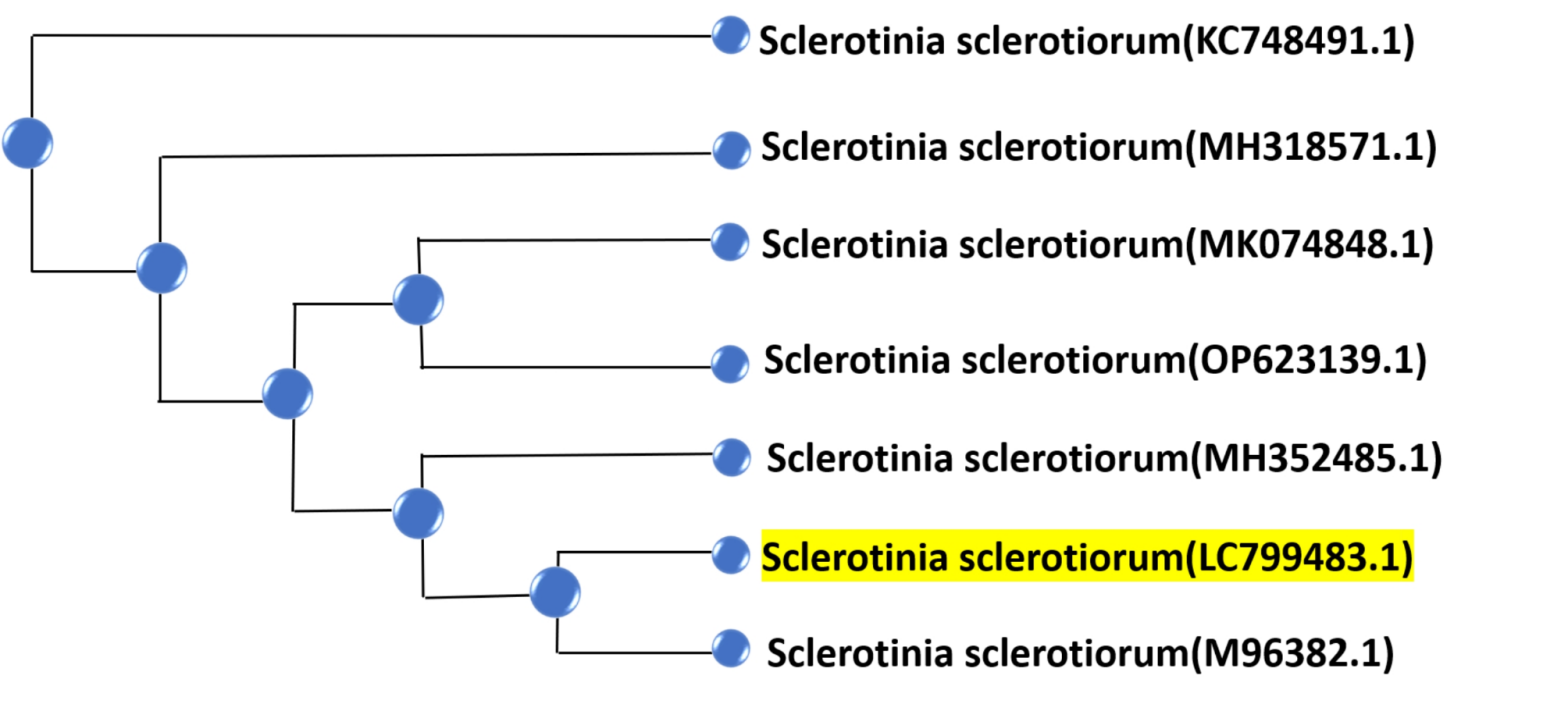
A neighbor-joining tree for the isolated *Sclerotinia sclerotiorum* strains. The *S. sclerotiorum* in this study was deposited in GeneBank with accession number LC799483.1.

The fungal ITS region’s PCR was sequenced and checked with the GenBank sequence databases. All obtained sequences were aligned and analyzed. The best-fitting substitution model for the alignment was the Kimura 2 parameter model with gamma distribution. A neighbor-joining tree was constructed based on the ITS sequence of all strains (Fig. 1). The ITS sequence gave high similarities with S. sclerotiorum, and the sequence had accession number LC799483.

### Optimization of the green synthesis of Se NPs

The preparation process and selection of the proper citrus peel were optimized to produce NPs with smaller particle sizes, higher stability, and dispersity. Typically, smaller NPs are known to have more potent antifungal activity than larger NPs^26–28^. The size distribution of Se NPs synthesized by lemon peel extract (L.P. -Se NPs) and orange peel extract (O.P. -Se NPs) was imaged by TEM. The charge of the nanostructure surface in both cases was detected using zeta potential analysis. Further characterizations of UV, FT-IR, and XRD spectroscopy were also performed to confirm and optimize the green synthesis of Se NPs.

**Fig. 2:**
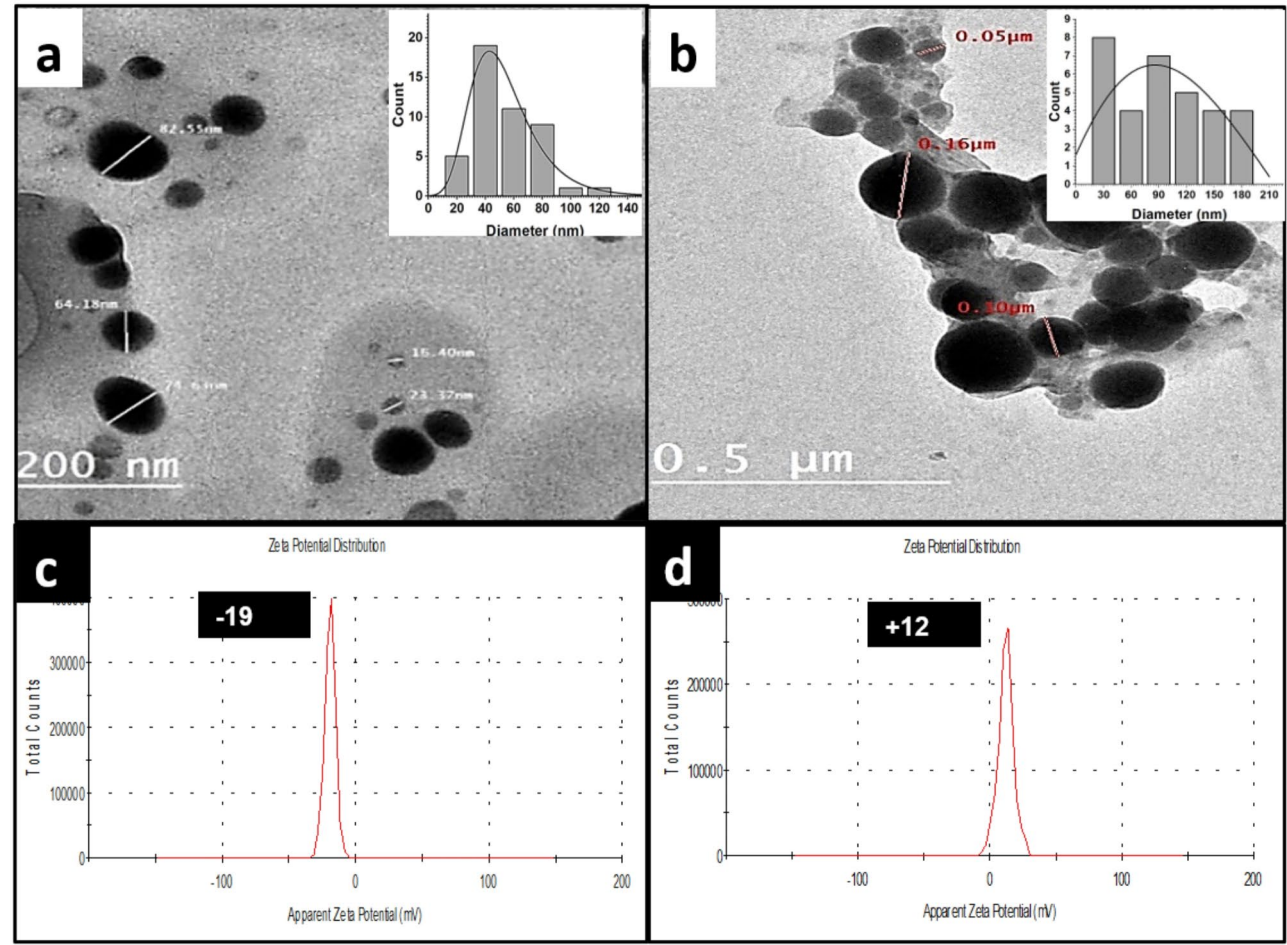
TEM Image of Se NPs synthesized by (**a**) lemon-peel extract (L.P.- Se NPs) with an inset histogram showing the peak diameter at 42.8±,18.5 nm (**b**) orange-peel extract (O.P.- Se NPs) with an inset histogram showing the peak diameter at 85.7±,140.22 nm. (**c** & **d**) represents the zeta potential and PDI for L.P.-Se NPs and O.P.- Se NPs, respectively.

Figure 2a, b shows TEM images for L.P. -Se NPs and O.P. -Se NPs, respectively, showing size distribution histogram (inset in each). The results confirmed the green synthesis of Se NPs with better size distribution of L.P. -Se NPs with no aggregations, as shown in Fig. 2a. In this case, the average diameter was 42.28 ± 18.5 nm, while the average diameter of O.P. -Se NPs was 85.7 ± 140.22 nm. The dispersion of both types of NPs was confirmed by measuring the polydispersity index (PDI) of both samples with a Zeta sizer. The results showed that (PDI) was better in the case of L.P. -Se NPs, with a value of 0.196 compared to 0.277 in the case of O.P. -Se NPs. It was previously studied that PDI values below 0.05 were mainly observed in samples with relatively uniform particle sizes. In contrast, PDI values exceeding 0.7 suggest a significantly wide range of particle size distribution^29^. Results for both samples show size ranges below 0.7, indicating the dispersity of NPs with better size distribution values for L.P. -Se NPs^30^.

Figure 2c, d presents the zeta potential of L.P. -Se NPs and O.P. -Se NPs to be -19 mv and + 12 mv, respectively. The zeta potential is the electrostatic potential when measured at the electrical double layer around a nanoparticle in solution. Neutral nanoparticles have a zeta potential between − 10 and + 10 mV, whereas strongly cationic and strongly anionic nanoparticles have zeta potentials more enormous than + 30 mV or less than − 30 mV^31^. Therefore, both zeta values indicate little colloidal stability that could be enhanced using a stabilizing agent^32^. However, L.P. -Se NPs exhibited higher stability than O.P. -Se NPs, distributed without aggregation and agglomeration. These results were aligned with previous studies of synthesized metallic NPs using Lemon peel extract^33^.

**Fig. 3:**
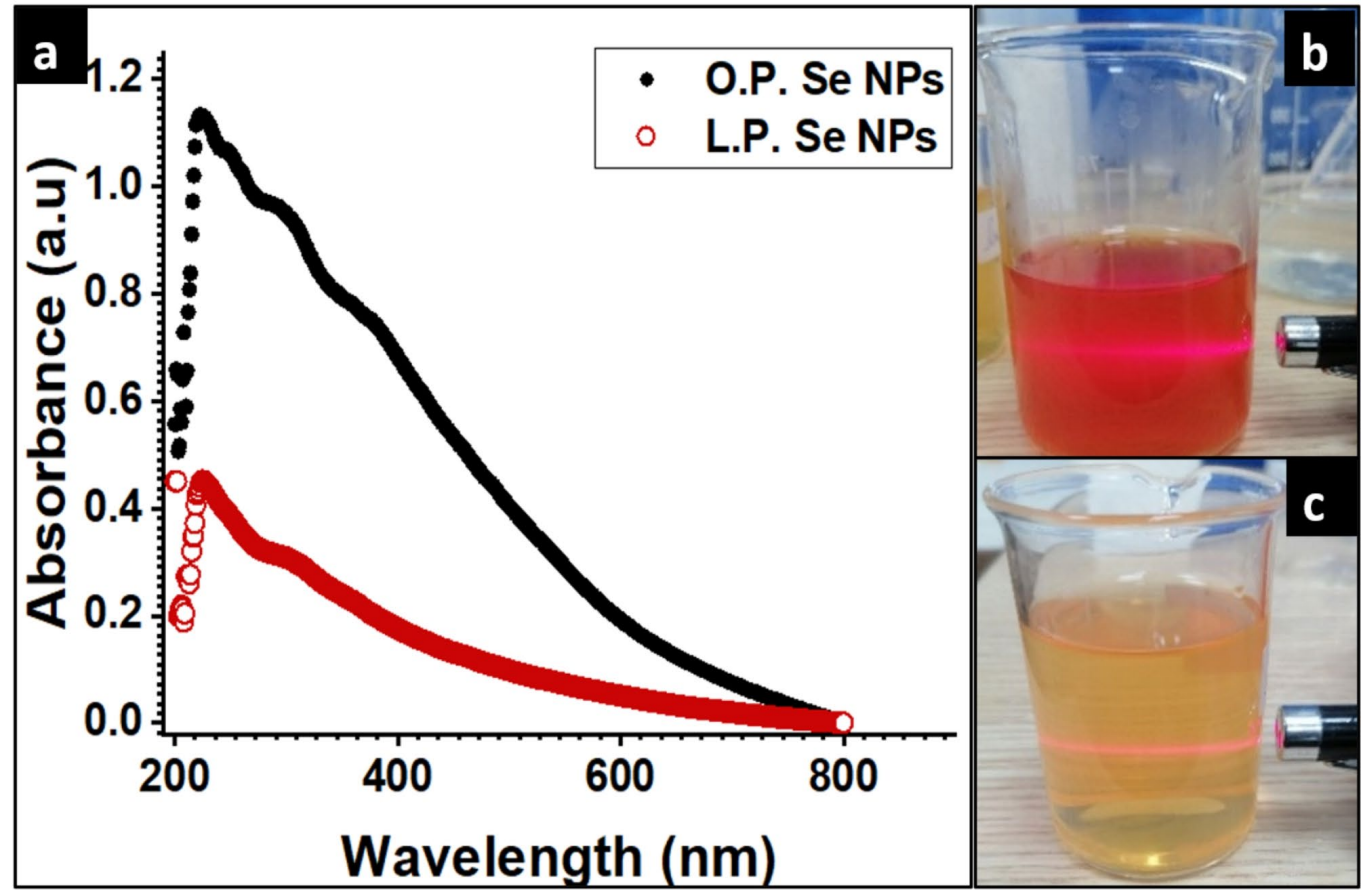
Shows the characterization of the synthesized NPs, (**a**) UV spectroscopy of green synthesized O.P.- Se NPs, and L.P.- Se NPs (**b** & **C**) shows the laser beam used to detect the NP preparation from both Orange and Lemon peels respectively.

As shown in Fig. 3a, the results of UV analysis revealed peaks that are displayed at 222 nm and 226 nm in the case of L.P. -Se NPs and O.P. -Se NPs, respectively; these peaks represent the formation of Se NPs due to the reduction of selenite ions by ascorbic acid found in lemon-peel and orange-peel extracts into elemental selenium (Se^0^)^34^. The UV-visible absorption maximum due to the surface plasmon resonance (SPR) of Se NPs was between 200 and 400 nm^35^. The significant blue shift in the absorption band of L.P. -Se NPs and O.P. -Se NPs was due to the biogenic origin of the synthesized Se NPs^36,37^. The little red shift in the absorption band of O.P. -Se NPs compared to those of L.P. -Se NPs indicates a larger particle size^37^.

As shown in Fig. 3b, c, a laser beam was used initially to confirm the formation of NPs in both cases due to the Tyndall effect. The Tyndall effect was tested to verify the colloidal and dispersed structure of nanoparticles^38^. The difference in colors between both samples reflects the particle size in both cases, as the solution of O.P.-Se NPs appears red compared to pale orange or yellow in the case of L.P. -Se NPs^39,40^. These color changes relate directly to the absorption features of the NPs in the visible spectra region and, consequently, their particle sizes. These results matches well with a previous study related to the size-dependent absorption of Se NPs^41^. The concentration of reducing agent in both extracts affects the nucleation and growth sequences as the higher concentration of ascorbic acid in lemon-peel extract results in smaller particle size^41^. This result agrees with previous studies, that found that lemon-peel extract contains the most elevated ascorbic acid compared to all other citrus-peel extracts^42,43^.

**Fig. 4:**
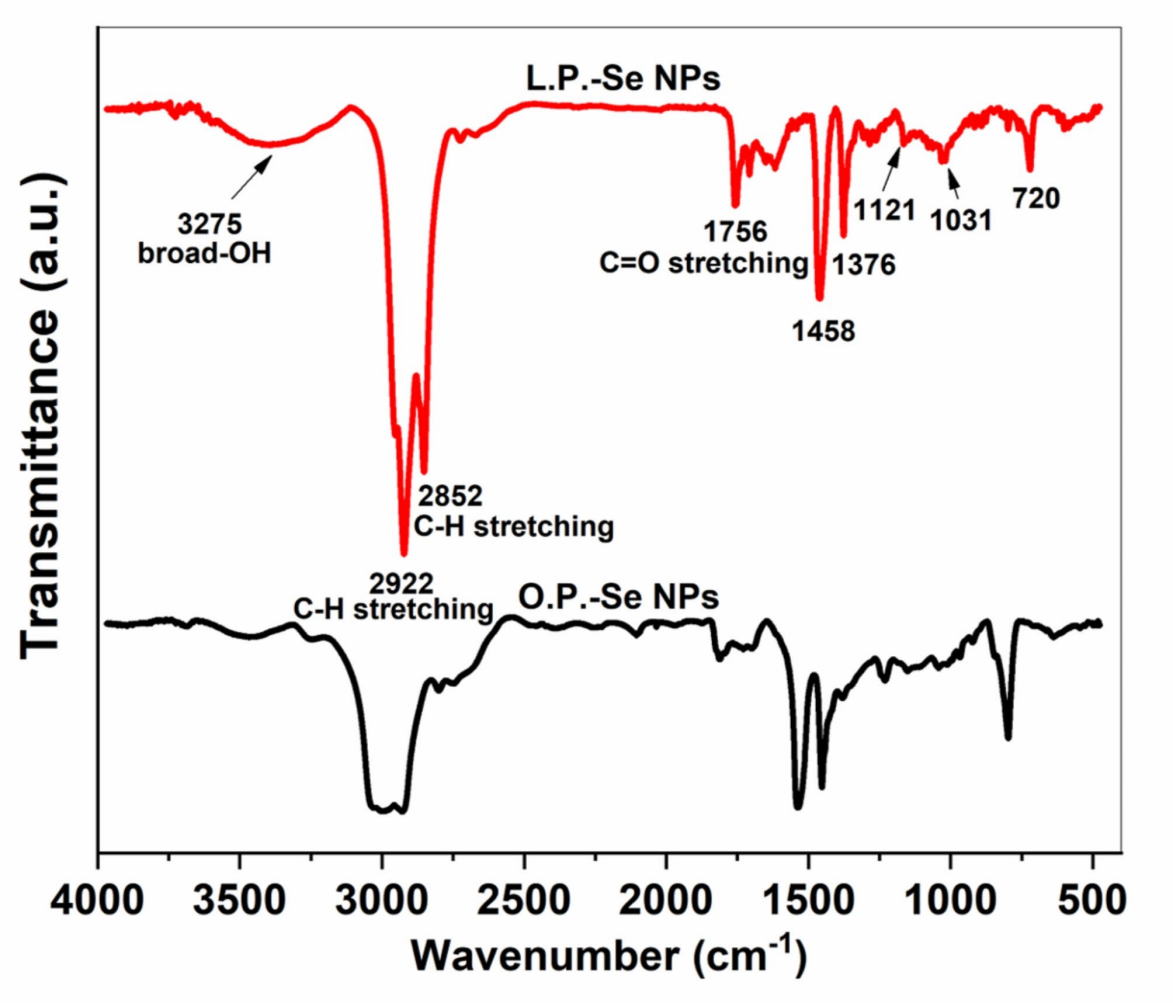
FT-IR spectroscopy of Se NPs synthesized by lemon and orange-peel extracts.

In Fig. 4, FT-IR analysis presented the green synthesis of Se NPs, using lemon or orange-peel extract, in the region from 400 cm^− 1^ to 1500 cm^− 1^. The observed multiple infra-red absorption bands are due to ionic bond vibrations found in Se metal. The 720 cm^− 1^ band represents the C-X stretching in alkyl halides. The 1121 cm^− 1^ and 1031 cm^− 1^ bands represent the C–N stretching of the amines, the 1376 cm^− 1^ band is related to the C– H bending in alkanes, a strong band at 1458 cm^− 1^ is noticed, which is due to the C-C Stretching occurred in the ring of aromatics. The diagnostic region involves a 1756 cm^− 1^ band related to C = O stretching in carboxylic acids. This enhanced band appears in the spectrum of L.P. -Se NPs more precise and sharper than those of O.P. -Se NPs, which may indicate the powerful reduction of SS in the case of lemon extract to form Se NPs compared to those in the case of orange extract^44^. Robust and sharp absorption bands at 2852 cm^− 1^ and 2922 cm^− 1^ correspond to C-H stretch alkynes. The broad O-H band observed at 3275 cm^− 1^ is associated with O-H stretching in alcohols; these absorption bands aligned with another study of biogenic Se NPs^45^.

As observed in Fig. 4, the intensity of most absorption bands in the case of L.P. -Se NPs is higher than O.P.-Se NP bands. The reason for this difference is due to the decrease in particle size and the increase in surface area to volume ratio, leading to higher intensity of absorption bands in the FT-IR spectrum as smaller particles have a more significant proportion of atoms on the surface, which can interact more readily with the incident infrared radiation, resulting in a stronger absorption signal^44^. Additionally, smaller particles may exhibit quantum confinement effects, leading to changes in the electronic and vibrational properties that can affect the intensity of absorption bands in the FT-IR spectrum. Hence, the FT-IR analysis results confirm the UV analysis results, indicating a lower particle size in the case of L.P. -Se NPs than in the case of O.P. -Se NPs^44,46^.

**Fig. 5:**
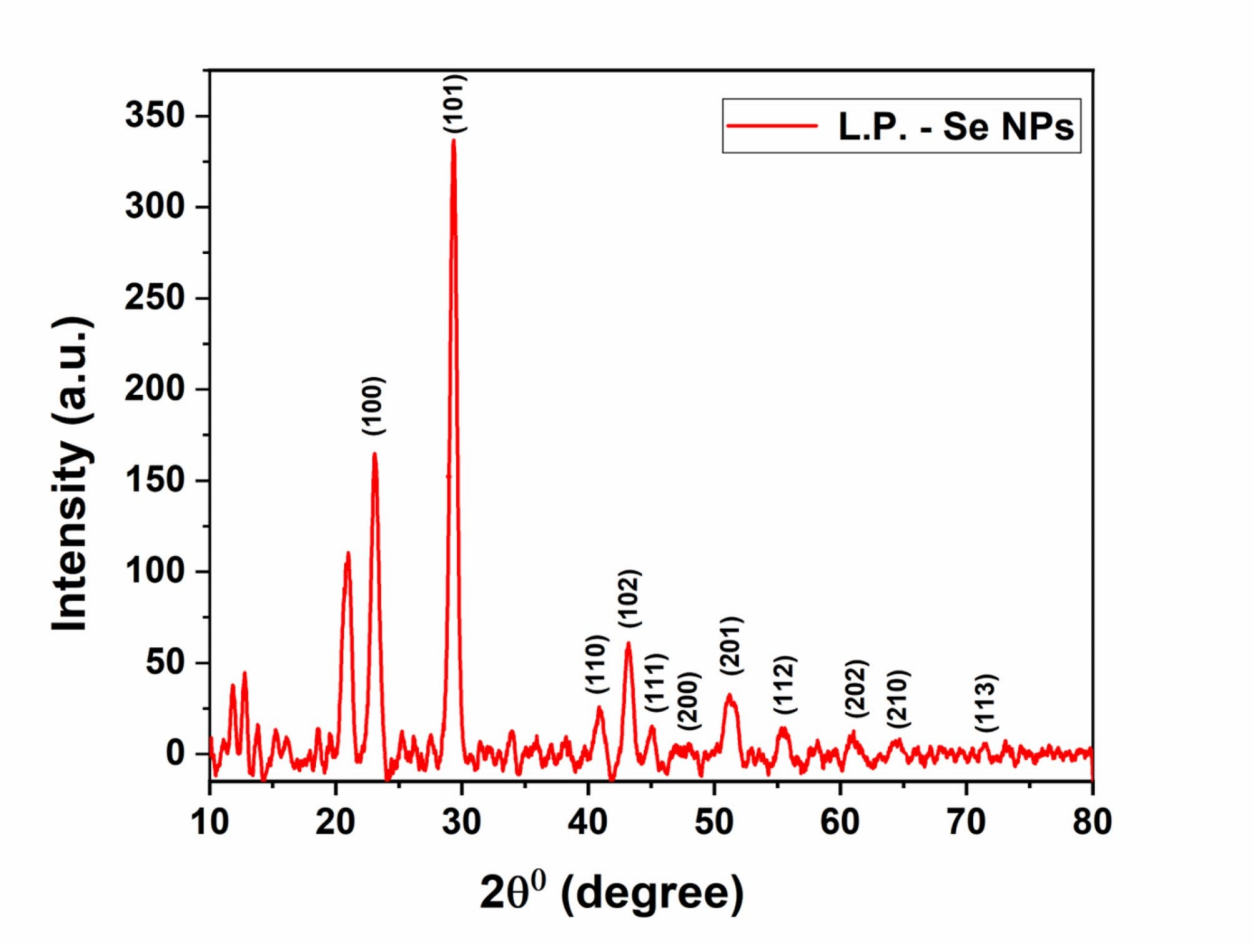
XRD pattern of L.P.-Se NPs.

The crystallinity of L.P. -Se NPs was further investigated using X-ray powder Diffraction (XRD) analysis. Figure 5 shows a typical XRD pattern of the triagonal structure of L.P. -Se NPs in which the diffraction peaks correspond to the following miller indices: (100), (101), (110), (102), (111), (200), (201), (112), (202), (210), (113). Some previous studies confirmed this result^47,48^. All these diffraction peaks are associated with the trigonal structure of Se NPs with an average crystallite size of 15.91 ± 0.98 nm. This Data agrees with those documented in the JCPDS standard card (No. 06–0362) for Se NPs.

The particle size of synthesized NPs is one of the most critical factors affecting the antimicrobial and antifungal activity of NPs. *Sribenjarat et al.*. noted that Se NPs with a smaller size range showed higher antimicrobial activity^49^. L.P. -Se NPs exhibit higher stability and dispersity relative to O.P. -Se NPs with lower particle size, which may be due to the higher content of ascorbic acid and other capping agents in lemon-peel extract, which are required for the reduction and stabilization of Se NPs^42,43,50^. However, a stabilizing agent could improve the little stability of negatively charged L.P. -Se NPs due to their low zeta value. Accordingly, negatively charged L.P. -Se NPs were selected in this study to be stabilized by cationic nano chitosan, which improves its stability and antifungal activity against the causal pathogen^51^.

### Preparation and characterization of CS NPs and NCS-Se NPs

Due to the number of amino groups on its surface, The cationic nature of chitosan is the reason for its positive zeta potential^52^. This cationic nature helps the coating process and stability of negatively charged L.P. -Se NPs to form NCS-Se NPs. CS NPs and NCS-Se NPs were prepared as described above in the method section.

**Fig. 6:**
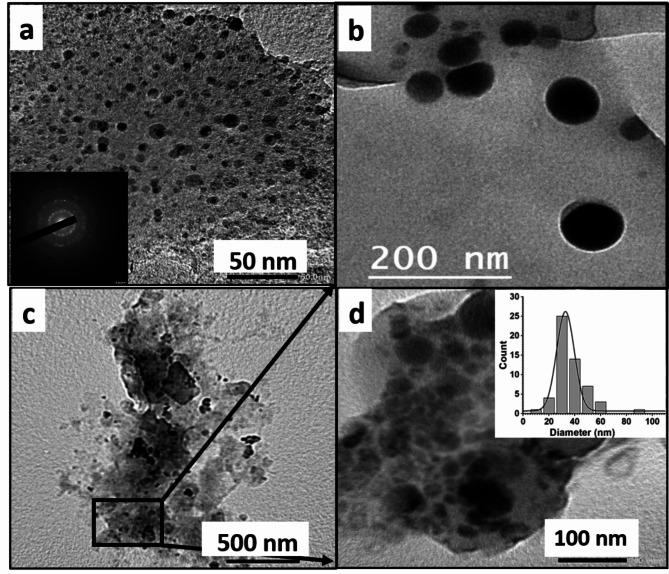
TEM images of (**a**) CS NPs with an inset showing the polycrystalline diffraction pattern of CS NPs, (**b**) Se NPs, (**c**) NCS-Se NPs, (**d**) zoom-in panel from (**C**) showing NCS-Se NPs with inset showing size distribution with peak Diameter of 32.7 ± 16 nm.

Figure 6a shows the TEM image of CS NPs with a tiny particle size of an average diameter of 6.43 ± 0.2 nm. The crystallinity of CS NPs was also investigated by the polycrystalline diffraction pattern (inset in Fig. 6a). Figure 6b exhibited a TEM image of the (uncoated) L.P. -Se NPs with small particle size and good dispersion due to the presence of extract biomolecules that serve as capping agents providing stabilization and reduced particle size with an average diameter of 42.8 ± 18.5 nm. However, the presence of CS NPs as a coating agent provided L.P. -Se NPs with further stability and produced (coated) L.P. -Se NPs with a smaller particle size of an average diameter of 32.7 ± 16 nm, as shown in TEM images (Fig. 6c, d). previous results confirmed the data obtained from TEM images^53–55^. The aggregation of particles in the presence of CS may be due to using high molecular weight CS. Higher and medium-molecular-weight CS is considered a flocculant that promotes particle agglomerations by inter-particle bridging^56^.

**Fig. 7:**
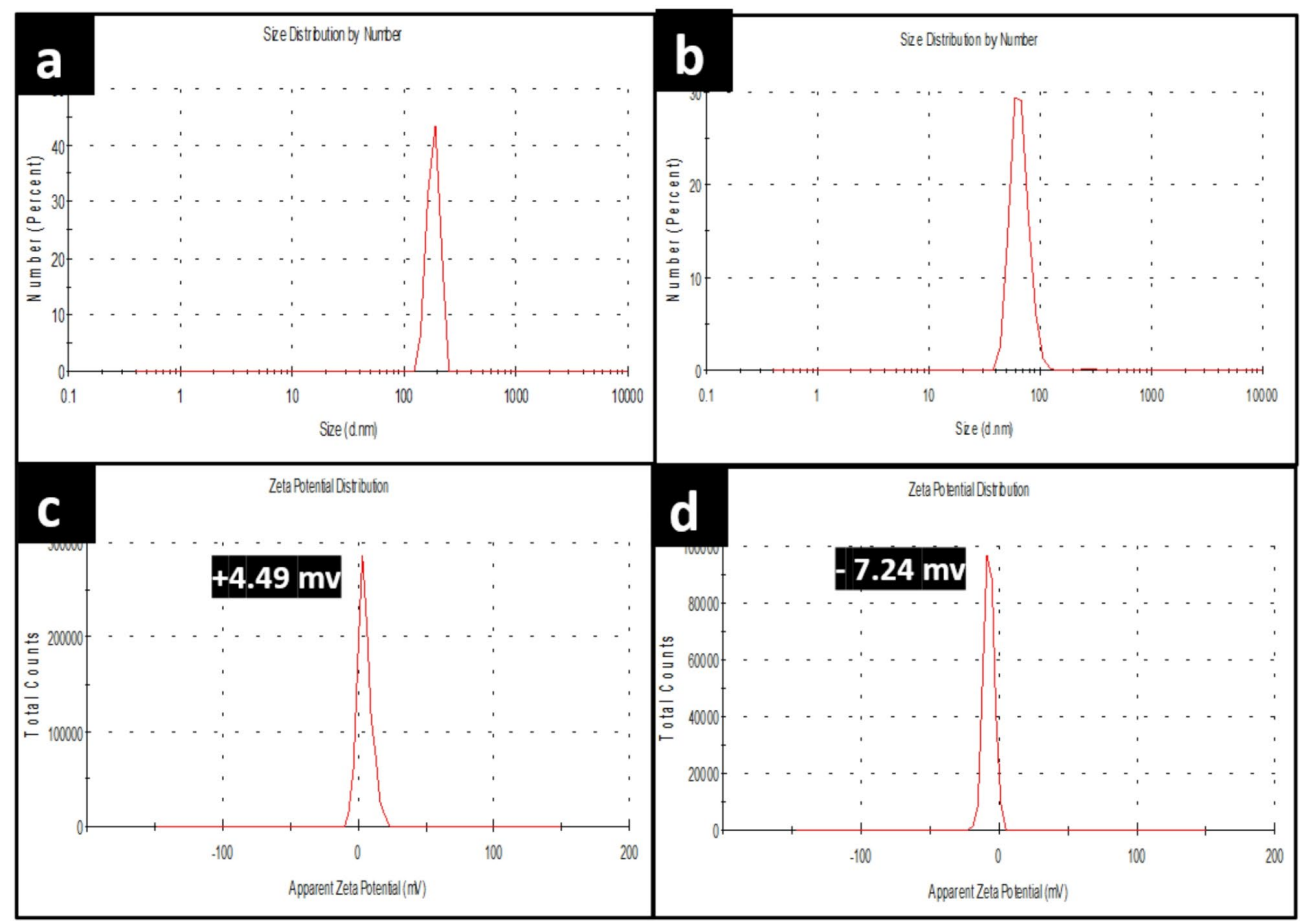
Size distribution of (**a**) CS NPs, (**b**) NCS-Se NPs, and zeta potential of (**c**) CS NPs, and (**d**) NCS-Se NPs, respectively.

Dynamic light scattering (DLS) technique has been used to measure the hydrodynamic particle size of prepared CS NPs and NCS-Se NPs. Figure 7a b shows 190 d.nm and 58.77 d.nm size peaks of CS NPs and NCS-Se NPs, respectively. The size measured by dynamic light scattering is significantly more than the actual size of nanoparticles Since DLS analyzes the particle’s hydrodynamic size in its aqueous state^17^. Figure 7c shows the zeta potential results for CS NPs to be + 4.49 mv. Coating of L.P. -Se NPs with CS NPs increases its zeta potential from − 19 mv (as shown in Fig. 2) to -7.24 mv due to the positive zeta potential of CS NPs (Fig. 7d), which indicates the efficient coating of CS NPs with Se NPs to form nanocomposite NCS-Se NPs. This data was confirmed by previous surface chemistry studies of Se-NPs after coating them with chitosan^57,58^ The estimated Particle size and zeta potentials of L.P. -Se NPs, O.P. -Se NPs, CS NPs, and NCS-Se NPs are illustrated in Table 1.

**Table 1:** Particle size distribution and zeta potential of biosynthesized selenium nanoparticles using lemon and orange-peel extracts (L.P.-Se, O.P.-Se), chitosan nanoparticles (CS), and their nanocomposite (NCS-Se).

Nanoparticles	Size Range (nm)	Mean Diameter (nm)	Zeta Potential (mv)
L.P.-Se	23.78–60.78	42.28	− 19.0
O.P.-Se	54.52–225.92	85.7	+ 12.0
CS	6.23–6.63	6.43	+ 4.49
NCS-Se	16.7–48.7	32.7	− 7.24

**Fig. 8:**
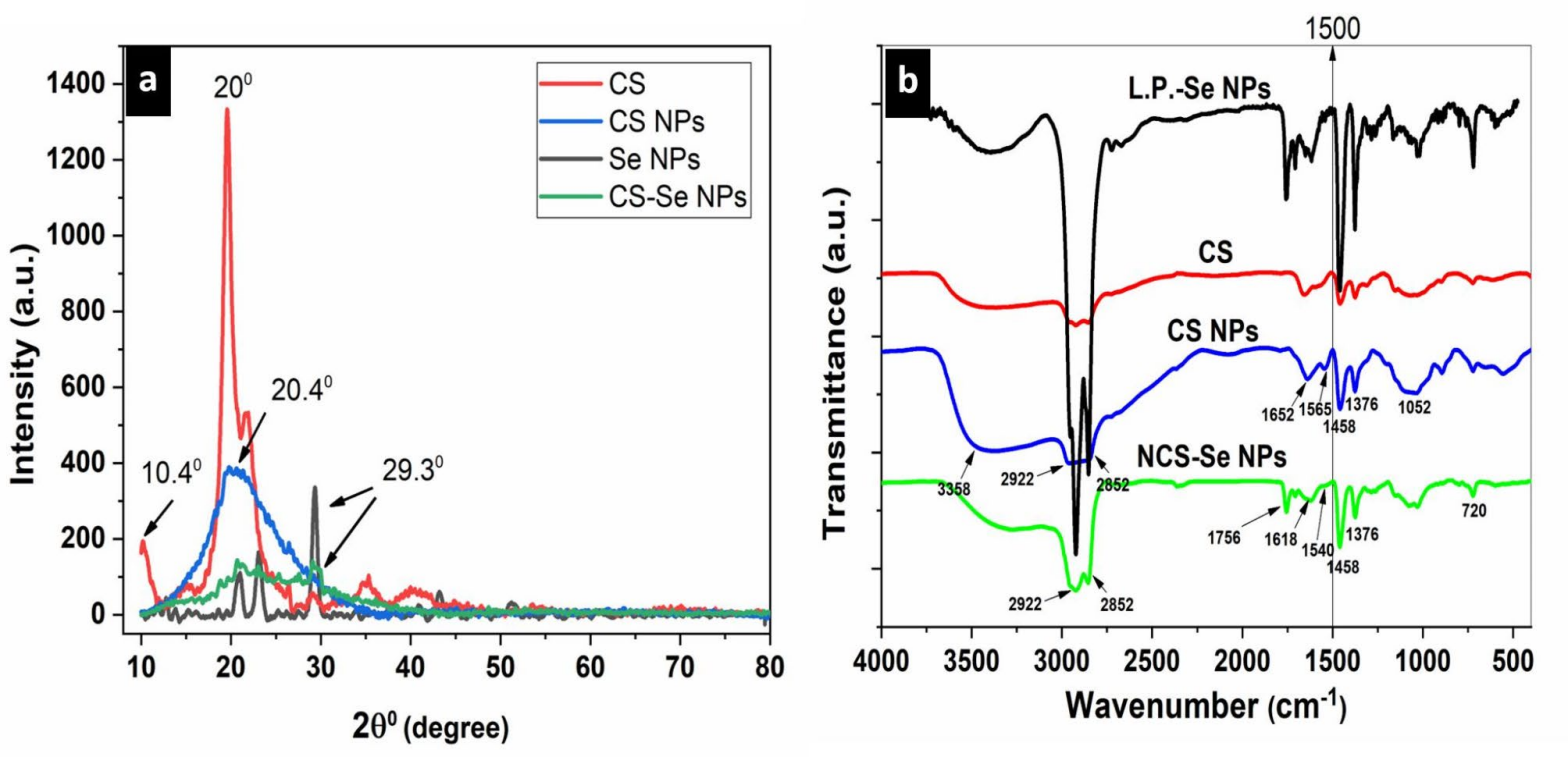
(**a**) XRD of CS, CS NPs, L.P.- Se NPs & NCS-Se NPs & (**b**) FT-IR of CS, CS NPs, L.P.-Se NPs & NCS-Se NPs.

Figure 8a shows the XRD pattern of CS, CS NPs, L.P. -Se NPs, and NCS-Se NPs. XRD pattern of CS NPs exhibits a broader prominent peak with lower intensity than (CS) at 2θ = 20.6°^59,60^. XRD pattern of NCS-Se NPs has two prominent peaks at 2θ = 20.6°, related to nano-chitosan, and 2θ = 29 °, associated with L.P. -Se NPs with lower intensities^47,48^. This result indicates the efficient coating of L.P. -Se NPs with CS NPs to form NCS-Se NPs. The well-dispersed nano-selenium in the nanocomposite matrix may cause the distinctive XRD peaks of Se NPs and CS NPs to overlap, and the appearance of additional peaks in the nanocomposite structure suggests that ionic interactions are influencing the crystal lattice. Previous studies confirmed these XRD results^61,62^.

Figure 8b shows the FT-IR pattern of CS, CS NPs, NCS-Se NPs, and L.P. -Se NPs to investigate the intermolecular interaction of nanoparticles. In nano-chitosan, several characterization peaks were noted. The first peak, at 3358 cm^− 1^, was assumed to represent an O-H stretch. Peaks at 1652 cm^− 1^ and 1565 cm^− 1^ represent C = O stretching from amide I, N–H bending, and C–N stretching from amide II, respectively. Additional 1458, 1376, and 1052 cm^− 1^ peaks were attributed respectively to skeletal vibration of C–O stretching, –CH_2_ bending, and –CH_3_ symmetrical deformation. These findings aligned with previous results, indicating the formation of CS NPs^63^. The electrostatic interaction between L.P. -Se NPs and the amino groups of NCS was confirmed by a shift in the absorption peaks of the NCS spectrum of amide I from 1652 cm^− 1^ to 1618 cm^− 1^ and amide II from 1565 cm^− 1^ to 1540 cm^− 1^ when compared to the spectrum of NCS-Se NPs implies that NCS might attach itself to L.P. -Se NPs by forming hydrogen bonds with -OH^64^. The intensity of absorption bands associated with L.P. -Se NPs at 2852 cm^− 1^ and 2922 cm^− 1^ decreased in the NCS-Se NPs spectrum due to the efficient coating of NCS with L.P. -Se NPs in its matrix^44^.

### In vitro antifungal activity

#### Mycelial growth assay

The antifungal activity of the three prepared nanostructures (CS NPs, L.P. -Se NPs, and NCS-Se NPs) had been studied compared to their bulk sources CS, SS, and a mixture of CS with SS (1:1 v/v) at the same concentrations (0.5,1,5,10,50,100 ppm) using the well diffusion method.

All calculated values in the above two tables are shown as mean ± SD (*n* = 3). Data within the groups were analyzed using one-way analysis of variance (ANOVA) followed by a, b, c, d, e, f Duncan’s multiple range test (DMRT), LSD = Least Significant Difference.

**Fig. 9:**
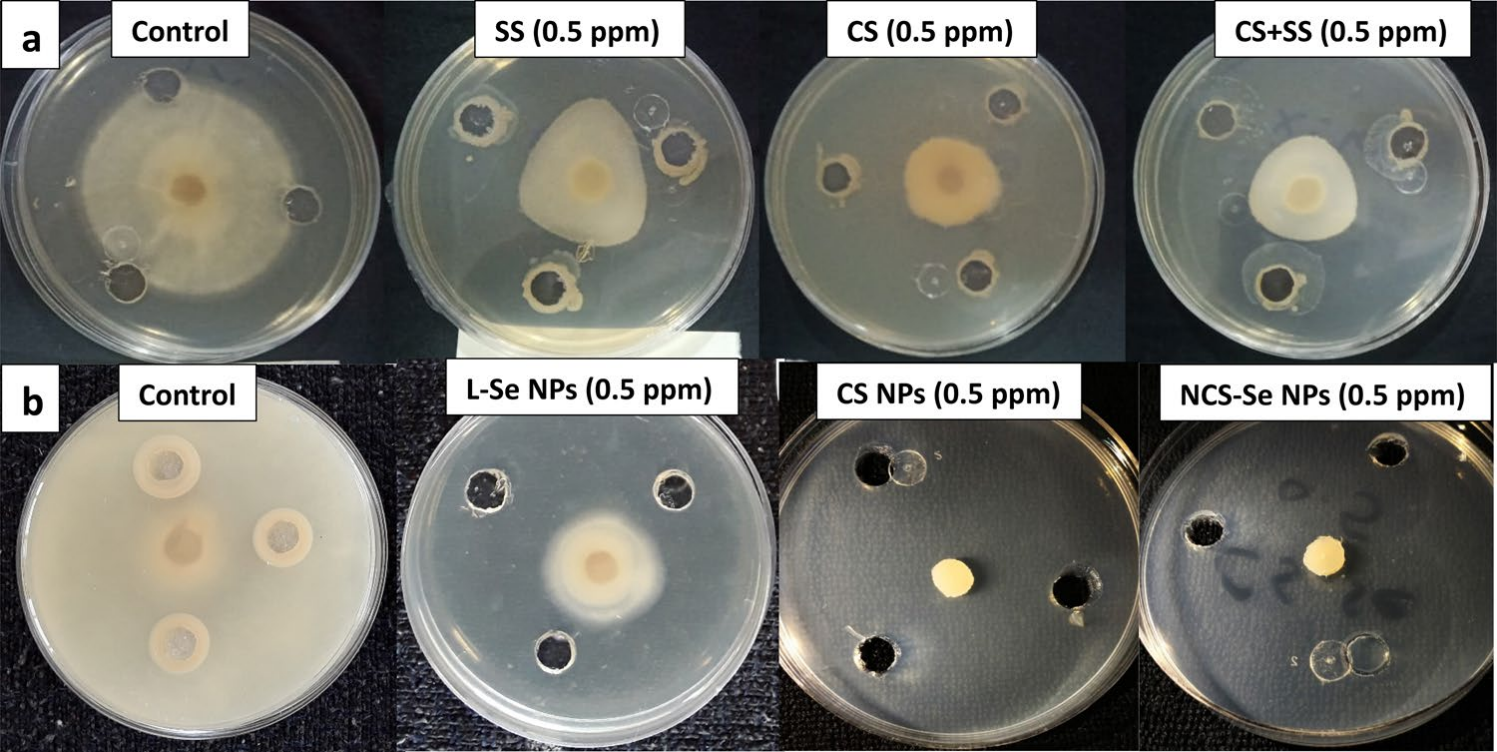
The mycelial growth of *S. sclerotiorum* treated with MIC of (**a**) SS, CS, and mixture of SS + CS (1:1 v/v) and (**b**) Se NPs, CS NPs, and NCS-Se NPs.

The results showed an antifungal activity of all bulk materials at all concentrations, as shown in Table 2, which agrees with previous reports on the antifungal activity of CS and SS against *S. sclerotiorum*^8,24^. The plasma membrane of *S. sclerotiorum* mycelia treated with this optimal inhibitory concentration of 1% and pH 5 of high molecular weight CS was shown to be significantly damaged^23^. The mycelial growth diameter of SS, CS, and their mixture at the MIC of 0.5 ppm were 3.69 ± 0.23,2.81 ± 0.11, and 3.09 ± 0.09 cm, respectively. Their calculated inhibitory percentage was 39.2%, 55.9%, and 50.6%, respectively (Fig. 9a).

**Table 2:** Growth diameter count of *S. sclerotiorum* treated with different concentrations of three tested bulk materials (SS, CS, and mixture of SS + CS (1:1 v/v)).

Concentration (ppm)	Growth Diameter (cm)		
	SS treatment	CS Treatment	SS + CS (1:1 v/v)
0	5.76^c^ ± 0.21	5.76^a^ ± 0.21	5.76^a^ ± 0.21
0.5	3.70^a^ ± 0.24	2.82^a^ ± 0.12	3.10^a^ ± 0.10
1	5.00^b^ ± 0.72	3.14^a^ ± 0.28	4.56^c, d^±0.22
5	3.67^a^ ± 0.16	3.10^a^ ± 0.02	3.59^a, b^±0.410
10	3.68^a^ ± 0.30	4.54^b^ ± 0.60	4.93^e^ ± 0.31
50	4.38^a, b^±0.40	3.00^a^ ± 0.31	4.10^b, c^±0.10
100	4.28^c^ ± 0.36	2.55^a^ ± 0.27	4.76^d^ ± 0.48
LSD	0.72	0.59	0.66

^a^,^b^,^c, d^,^e^,^f^ These letters are for Duncan’s multiple test, which means the treatments with the same letter are non-significantlydifferent but the treatments with different letters are significantly different.

As shown in Table 3, among all inhibitory concentrations, the MIC of the three nanostructures was also at minimum concentrations of 0.5 ppm with inhibitory percentages of 69%,100%, and 100%, respectively (Fig. 9b). The antifungal effect of LP- Se NPs at a low concentration of 0.5 ppm with 69% inhibitory percentage was significantly supported after coating with CS NPs. The 100% inhibitory percentage was obtained by NCS-Se NPs, as CS NPs serve as a stabilizing agent, besides their strong antifungal effect, promoting the antifungal activity of green synthesized Se NPs. This finding agrees with a previous study^14^, which found that chemically synthesized Se NPs with Poly-L-Lysine (PLL.P. -Se NPs) can significantly inhibit the growth of *S. sclerotiorum* but at a maximum concentration of 100 ppm.

**Table 3:** Growth diameter count of *S. sclerotiorum* treated with different concentrations of three tested nanostructures (Se NPs, CS NPs, and NCS-Se NPs).

Concentration (ppm)	Growth Diameter (cm)		
	Se NPs treatment	CS NPs Treatment	NCS-Se NPs Treatment
0	8.98^c^ ± 0.03	8.98^f^ ± 0.03	8.98^d^ ± 0.03
0.5	3.13^a, b^±0.17	N/A	N/A
1	3.18^a, b^±0.25	4.59^b^ ± 0.08	N/A
5	3.10^a^ ± 0.16	5.64^c^ ± 0.40	6.77^c^ ± 0.62
10	3.22^a, b^±0.24	5.76^c^ ± 0.40	6.13^b^ ± 0.25
50	3.68^b^ ± 0.50	6.39^d^ ± 0.14	6.05^b^ ± 0.23
100	3.31^a, b^±0.46	7.22^e^ ± 0.49	6.33^b, c^±0.28
LSD	0.56	0.63	0.65

^a^,^b^,^c, d^,^e^,^f^ These letters are for Duncan’s multiple test, which means the treatments with the same letter are non-significantlydifferent but the treatments with different letters are significantly different.

#### Mycelial biomass assay

Freshly prepared fungal biomass treated with each tested nanostructure with the MIC of 0.5 ppm, besides control, were dried and weighed after five days of dynamic incubation using an orbital shaker at 25 °C and 180 rpm. The results exhibited potent inhibition of the tested pathogen growth, specifically in the case of NCS-Se NPs with the lowest average biomass at 0.32 ± 0.05 g compared to the average fungal biomass of other nanostructures and control, as shown in (Table 4). This result reflects the effectiveness of NCS-Se NPs as an eco-friendly antifungal agent against *S. sclerotiorum*.

**Table 4:** Dry weight yield of *S. sclerotiorum* treated with 0.5 ppm of CS NPs, Se NPs, and NCS-Se NPs.

Dynamic incubation (180 rpm)	Treatment concentration (0.5 ppm)			
	Control	CS NPs	Se NPs	NCS-Se NPs
Average biomass (g)	2.22 ± 0.04	1.00 ± 0.21	0.55 ± 0.16	0.32 ± 0.05

#### Morphological changes in S. sclerotiorum hyphae

**Fig. 10:**
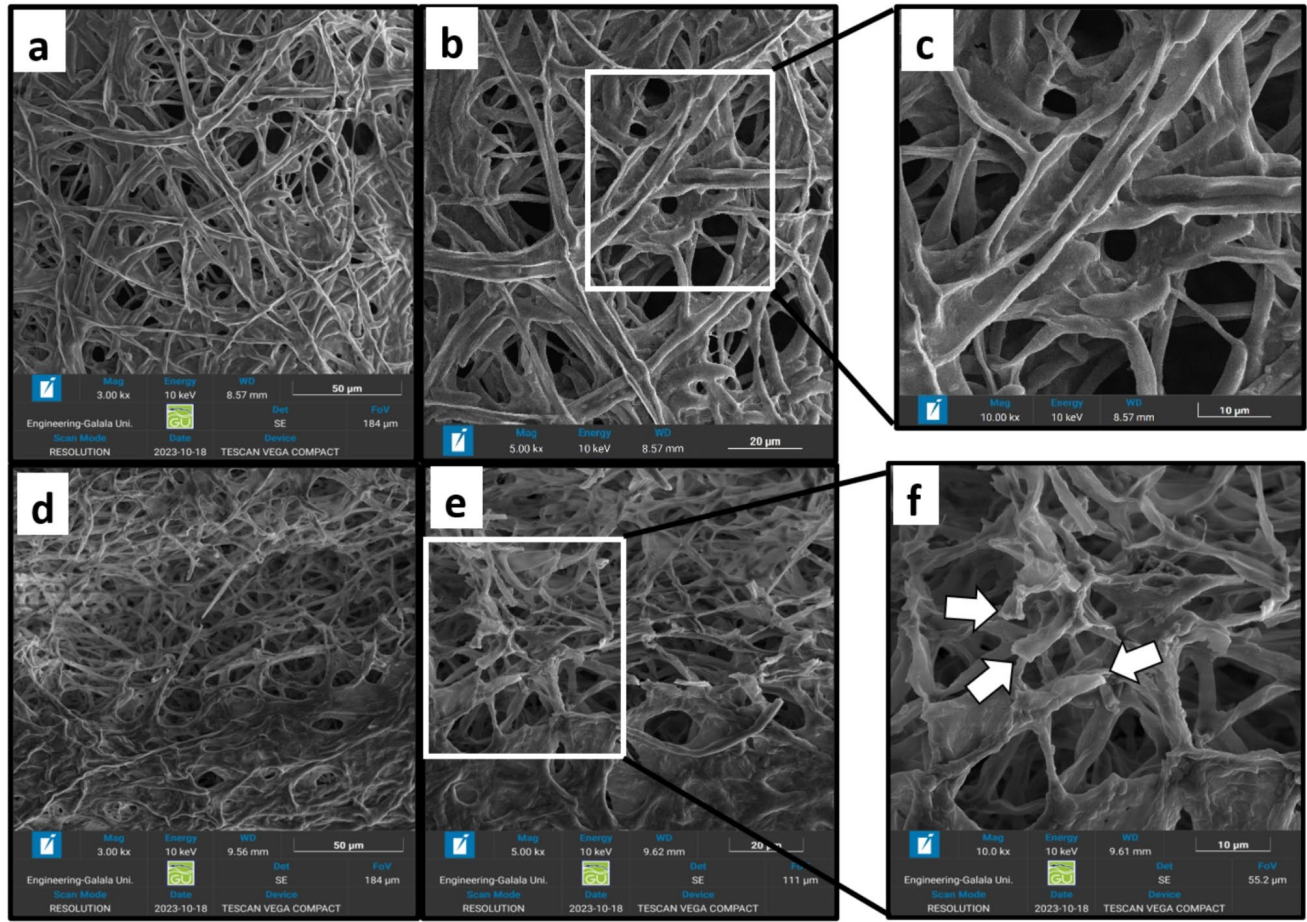
TEM images of *S. sclerotiorum* hyphae of (**a**-**c**): control micrographs and (**d**-**f**): 0.5 ppm of NCS-Se NPs treatment micrographs.

The Ultrastructural changes in the hyphae of *S. sclerotiorum* caused by 0.5 ppm of NCS-Se NPs were detected using a scanning electron microscope (SEM). The untreated hyphae of *S. sclerotiorum* appeared intact, dense, long, and cylindrical, as shown in Fig. 10a, b, c. In contrast, hyphae treated with 0.5 ppm NCS-Se NPs showed deformities and abnormalities in their morphology. These morphological changes include shrinkage, plasmolysis, distortion, and breakdown of fungal hyphae, as shown in Fig. 10d, e, f, and therefore, rupture and damage of *S. sclerotiorum* hyphae, which indicates the ability of our nanocomposite to suppress their vital functions.

### Antifungal mechanism of NCS-Se NPs

**Fig. 11:**
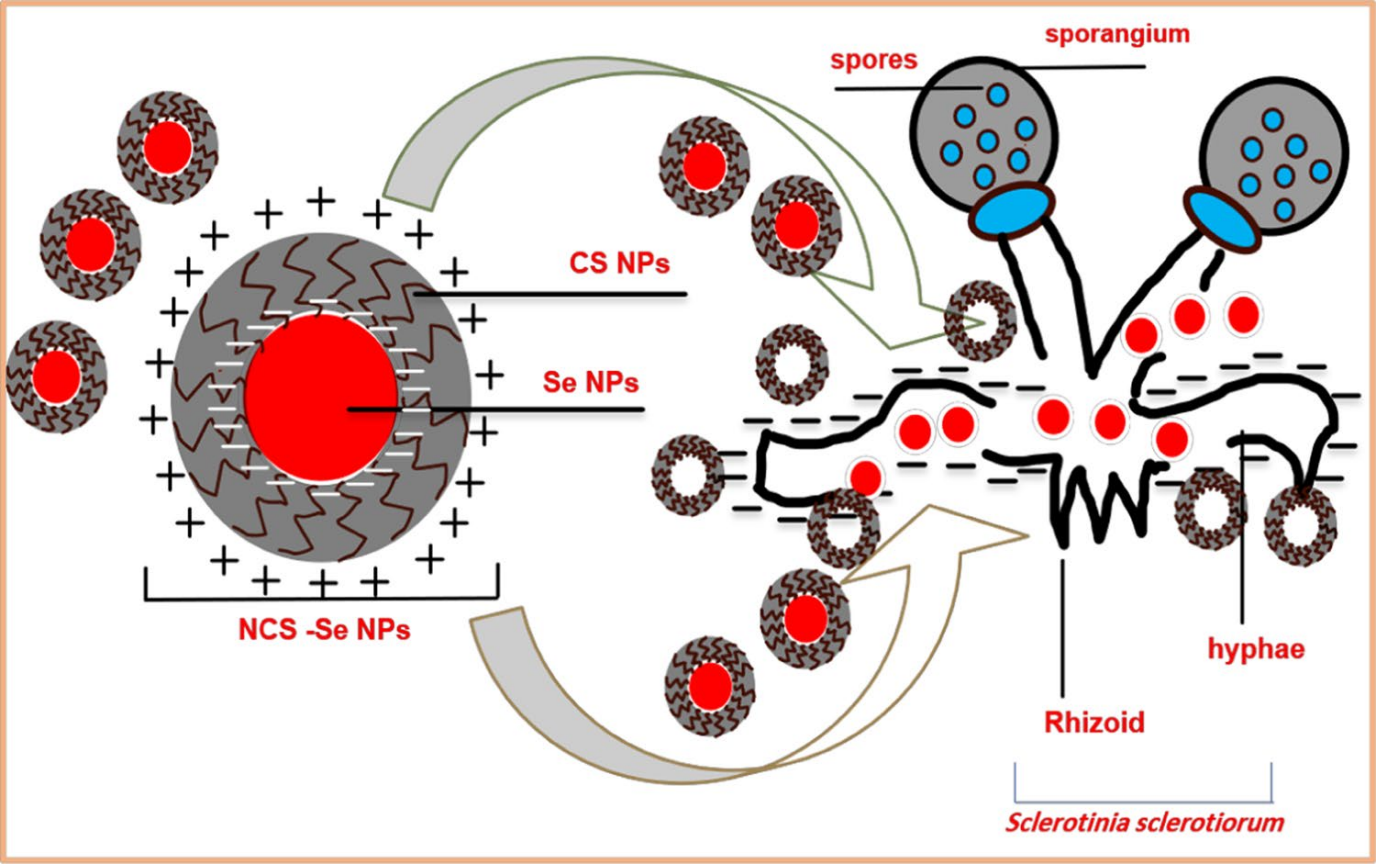
Schematic diagram showing the adsorption of NCS-Se NPs with *S. sclerotiorum* mycelia and their damage effect.

It has been demonstrated that CS and Se can protect plants from fungal infections^8,9,23^. In the current investigation, we discovered that the mycelial growth of ***S. sclerotium*** was significantly inhibited by MIC of NCS-Se NPs (0.5 ppm), which also alters the organism’s shape and damages its hyphae. This impact could result from the interaction of the positive amino groups in the nanocomposite’s CS coat with the negatively charged macromolecule residues on the fungal cell membrane, altering the plasma membrane’s permeability^23,65^. Following that, as shown in Fig. 11, the tiny CS NPs and some released green synthesized L.P. -Se NPs could enter the hypha and interact with intracellular organelles/bio-systems to inhibit their essential functions, which consequently lead to fungal deformation and lysis^50,66^. Moreover, liberated L.P. -Se NPs may improve the release of *S. sclerotiorum* electrolytes^67^. The changes in osmolyte levels of *S. sclerotiorum* mycelia may be necessary for the organism to adapt to its surroundings and protect against Se toxicity, leading to membrane system damage and metabolic disorder^8,9^. More information is needed on the protective effect’s mechanism, which must be investigated. However, identifying the antifungal mechanism that NCS-Se NPs caused to *S. sclerotiorum* mycelia would aid in developing an efficient, safe, and sustainable strategy for controlling and managing *S. sclerotiorum*.

## Materials and methods

### Chemicals

Sodium selenite anhydrous AR was purchased from SDFCL Co., India. High molecular weight Chitosan of 350 KD and Deacetylation level of 90% was purchased from Hunan Yun Bang Pharmacy Co., China. Sodium tripolyphosphate (TPP), sodium hydroxide pellets, and concentrated hydrochloric acid 32–35% were purchased from Sigma Aldrich Co., America. Potato Dextrose Agar (PDA) and potato dextrose broth (PDB) were purchased from Merck Co., Germany. All chemicals were directly used without any further purification.

### Isolation and identification of fungal strain

A common fungal disease called *Sclerotinia* rot, sometimes called white mould on some crops, is brought on by phytopathogenic members of the *Sclerotinia* genus. Specifically, *S. sclerotiorum* is regarded as one of the most pernicious and widespread plant infections. Necrotrophic fungal pathogen *S. sclerotiorum* (Lib.) de Bary causes diseases in various plants^68,69^. The fungus was isolated from diseased kidney bean legumes, such as cottony mycelium, on potato dextrose agar (PDA). The fungus was identified morphologically and microscopically and confirmed by molecular identification using ITS. The obtained sequence was deposited at NCBI for accession number. The ITS region was sequenced, and DNA was isolated from the fungal isolate to facilitate molecular identification.

A mixer mill isolation process recovered 0.5 g of fungal mycelium after five days of incubation. DNA was extracted, and a triplicate polymerase chain reaction (PCR) was conducted. 1x PCR buffer, 1.5 mM MgSO_4_, 2 mM dNTP mixture, 1 µM of each primer (ITS-F 5′-TCCTCCGCTTATTGATATGC-3′, ITS-R 5′-GGAAGTAAAAGTCGTAA CAAGG-3′), 1 µl of *Pfu* DNA polymerase (Fermentas, St. Leon-Rot, Germany), and 1 ng of template DNA were added to the 25 µl volume used for the reaction. The PCR amplification process involved an initial denaturation at 95 °C for 5 min, 25 cycles of 94 °C for 1 min, 55 °C for annealing for 45 s, and 72 °C for an extension lasting 45 s each. Purified and sequenced were the PCR products generated from the fungal isolate that showed up as a single band. The GenBank sequencing databases were then consulted. In MEGA5, the acquired sequence was aligned and examined. The Kimura 2 parameter model with gamma distribution was the best-fitting replacement model for the alignment. A maximum-likelihood tree was built based on the ITS sequence and the 1000 bootstrap values provided at the nodes.

### Green synthesis of Se NPs

*Citrus limon* (lemon) and *Citrus reticulata* (orange) are the citrus peel extracts selected, as both contain the highest ascorbic acid content^42,70^. All the fruits were purchased from the local market. Samples of citrus fruits were cleaned with double distilled water and dried with sterilized paper tissues. The dried citrus fruits were peeled, and the peels were cut into tiny pieces using a clean knife. 50 ± 0.005 g of citrus peel pieces was weighed accurately using a sensitive digital balance and stored in sterile conditions. The weighed peels were then ground in a mortar and turned into a fine mixture with a wetted appearance. The mixture was then boiled in 150 ml of double distilled water for 15 min until the color of the water changed into green-yellow, then filtered using Whatman Filter paper no.1^18^.

50 ml of the freshly prepared citrus peel extract (lemon or orange-peel extract was precisely heated using a magnetic hot plate stirrer, and conditions were adjusted to 40ºC and pH 4. 5 ml of (0.1 M) Sodium selenite (Na_2_SeO_3_) was added immediately. The formation of Se NPs was confirmed by forming a solution with an orange-red color at this optimum condition. The mixture was centrifuged at 10,000 rpm for 30 min, washed three times with double-distilled water, and a final wash with ethanol, dried, and stored at room temperature^18,71^.

### Preparation of CS solution and CS NPs

#### Preparation of CS solution

About 90% deacetylation level and 350 KDa average molecular weight of CS powder was used to prepare stock concentration of 1% (w/v) CS solution in 1% HCl by stirring overnight at room temperature until the whole amount was completely dissolved. HCl (1 N) and NaOH (1 N) were used to adjust the solution at pH 5.

#### Preparation of CS NPs

CS NPs were prepared using the ionotropic gelation method between CS and TPP^72^ Briefly, TPP was dissolved in double-distilled water to a concentration of 0.1% (w/v) under magnetic stirring at room temperature. Then 10 ml of TPP solution was added dropwise with the flow rate of 0.25 ml/min to 50 ml of the diluted previously prepared stock concentration of 0.1% (w/v) CS solution with a ratio 1:5 respectively, the pH level of the CS and TPP solutions was adjusted to pH 5 and pH 2 respectively. The mixture was stirred at 600 rpm for 30 min. Finally, NCS was collected by centrifugation (at 11.250×g for 28 min)^25,53^, washed three times with double distilled water, oven-dried at 50 ^0^C overnight, and stored at room temperature.

### Synthesis of NCS- Se NPs

NCS-Se NPs were green synthesized as Se NPs as described before but in the presence of CS, with some modifications described by *Shao et al.*^58^, *Zeng et al.*^55^, and *Ayoub et al.*^61^. The mixture of (CS-SS) with a ratio of 1:1 v/v was stirred at 1000 rpm for 30 min and added dropwise to freshly prepared citrus peel extract. The solution was left overnight with gentle stirring. After coating Se NPs with CS, 1 ml of 0.1% TPP cross-linker was added dropwise for ionotropic gelation. The color of the solution then changes from yellow to reddish-orange, which confirms the formation of NCS-Se NPs. The unreacted CS, Na_2_SeO_3_, and TPP are removed by dialysis (MWCO: 8–12 k Da) for 48 h at 4 °C, and a solution of NCS-Se NPs is obtained.

### In vitro studies of antifungal activity

Antifungal activity of the bulk materials (CS, SS, and a mixture of both materials with a ratio 1:1 v: v was evaluated against *S. sclerotiorum* at different concentrations (0.5,1,5,10,50 and 100 mg/L), then, the antifungal activity of the tested nanomaterials ( Se NPs, CS NPs, and NCS-Se NPs ) was also evaluated at the same concentrations via the healthy diffusion method to determine the minimum inhibitory concentration (MIC) of each tested material^14^. Finally, fresh mycelial biomass was assayed at the MIC to evaluate the inhibitory effect for each nanomaterial individually as well as the nanocomposite’s synergistic inhibitory effect.

#### Mycelial growth measurement

*S. sclerotiorum* was isolated from the infected green bean and kept on potato dextrose agar (PDA). According to Qing and Yao^23,73^, the impact of each previously prepared material on mycelial growth was evaluated. Briefly, 20 ml of sterilized PDA was prepared and placed in sterilized plates with a 9 cm diameter. Then, Mycelial disks with five mm-diameter from old cultures (10 days grown at room temperature) were placed in the center of each plate, three holes were made using sterilized tips in each sterilized PDA around each disk, and 1 ml solution of each prepared concentration in aseptic conditions was withdrawn and distributed in the 3 holes. Each treatment was replicated three times, and the experiment was repeated twice. PDA without any concentration served as a control. Control and treated plates were incubated at 25 °C for 5 days. After incubation, the mycelial growth was determined by measuring the growth diameter of each tested concentration; the antifungal activity of all tested concentrations on the mycelial growth of *S. sclerotiorum* was calculated according to the following formula:

$$Mycelial~growth~inhibition~rate~\left( \% \right)~ = \frac{{Growth~Diameter~of~Control - ~Growth~Diameter~of~Treatment}}{{Growth~Diameter~of~Control~ - ~\left( {0.5~cm} \right)}} \times 100$$

#### Mycelial biomass assay

100 ml of potato dextrose broth (PDB) was prepared and placed in a 250 ml conical flask. Each PDP was diluted by the MIC of each tested nanostructure (Se NPs, NCS, and NCS-Se NPs) until the tested concentrations were obtained. Then, mycelial disks with 5 mm- diameter from old cultures (10 days grown at room temperature) were inserted into each treated PDB at 180 rpm 25^o^C. Each treatment was replicated 3 times, and the experiment was repeated twice PDB without any concentrations served as a control. After collecting the fungal biomass, it was washed thrice with double-distilled water and left to dry on filter paper for two hours. Then, the fresh fungal biomass of each treatment and control group was dried and weighed after five days of incubation.

#### Morphological changes in S. sclerotiorum hyphae

After 18 h of exposure to the minimum inhibitory concentration (MIC) of NCS-Se nanoparticles (NPs), S. sclerotiorum hyphae were fixed with a 2.5% glutaraldehyde solution and washed three times for 10 min with 100 mM phosphate buffer. The fixed hyphae were then post-fixed for three hours in osmium tetroxide (1%) and dehydrated through an ethanol gradient to examine the ultrastructural changes in the hyphae using a scanning electron microscope (SEM) after coating with gold^74^.

### Characterization of the prepared nanostructures

#### Transmission electron microscopy (TEM)

The formation of the following nanostructures, green synthesized Se NPs, CS NPs, and NCS-Se NPs was confirmed using a Transmission electron microscope (TEM) (JEM-2100 PLUS). At an accelerating voltage of 200 KV, TEM was used to study the size, shape, and agglomeration states of Se NPs, CS NPs, and NCS-Se NPs. A drop of the diluted samples was placed on a copper-coated carbon grid and allowed to dry for about 15 min. A filter paper was used to remove the excess sample, and then the grid was left in the air to dry before being introduced to the TEM.

#### Dynamic light scattering (DLS)

The particle size of all prepared nanostructures, Se NPs, CS NPs, and NCS-Se NPs was measured using the Zeta sizer nano series (ZEN3600, Malvern, UK) with a size range (of 0.6:6000 nm) at 25 °C. Samples were diluted with deionized water before being introduced to the zeta sizer to measure their average diameter and size distribution. The device was outfitted using a He–Ne laser radiation beam at 633 nm, and the sample suspension was placed in a capillary cuvette. A photomultiplier tube detected the back-scattered light, and the samples’ average size was calculated. DLS also gives information about the surface charges (Zeta Potential) measured using the same capillary cuvette directly after measuring the particle size at the zeta potential range (-200:200 mV).

#### X-ray diffraction (XRD) analysis

X-ray diffraction analysis (XRD) was done to study the crystal structure, composition, and grain size of nanostructures using an X-ray diffractometer (Angstrom -ADX8000 diffractometer) with CuKa1-radiation source of wavelength 1.54060 A^0^ and two theta angles from 100 to 800.

#### Ultraviolet-visible (Uv-Vis) spectroscopy

The absorption spectrum of all prepared nanostructures was determined using UV-Vis. Spectrophotometer (BioWave3-USA) with a wavelength range of 200–800 nm at a resolution of 1 nm.

#### Fourier-transform infrared (FTIR) spectroscopy

Further characterization involved Fourier transform infrared spectroscopy (Nicolet™ iS50 FTIR Spectrometer in KBr with absorption in range (400–4000 cm^-1^) with resolution 1 cm^-1^ was used to evaluate the functional groups involved in the formation of green synthesized Se NPs and after coating with nano chitosan. The device was continuously purged with dry air to remove the water vapor from the atmosphere. FTIR helps to observe apparent changes in the structure of the compound by studying the variations of bandwidth and wavelength as well as the intensity and frequency of the vibration modes.

#### Scanning electron microscopy (SEM)

The morphological changes in the hyphae of the pathogen after treatment with the nanostructures were observed through SEM imaging (TESCAN VEGA COMPACT, Brno, Kohoutovice, Czech) at an accelerating voltage of 10 KeV.

### Statistical analysis

Statistical analysis of the collected data was performed via the ONE-WAY ANOVA analysis (at *P* < 0.05), Duncan’s multiple range states, and the least significant difference (LSD) statement ^75^. The results and data obtained were examined using the SPSS software version.

## Conclusion

Generally, we concluded that L.P.-Se NPs exhibit smaller sizes of 42.28 ± 18.5 nm and a better distribution than O.P.-Se NPs. The lower negative zeta value of L.P.-Se NPs and the smaller particle size enhanced their selection for coating with positively charged nano-chitosan to improve their stability and antifungal efficacy against *S. sclerotiorum*. CS NPs exhibited small particle sizes of 6.43 ± 0.2 nm with positive zeta value. This cationic nature and small particle size enhanced the properties of CS NPs and improved their antifungal activity against the same causal pathogen with a 100% inhibitory Percentage. Low zeta values of L.P.-Se NPs and CS NPs of -19 mv and + 4.49 mv, respectively, limited their stability and bioactivity. Combining NCS with L.P.-Se NPs to form NCS-Se NPs reduced its particle size with an average diameter of 32.7 ± 16 nm and increased its stability and antifungal activity. The antifungal activity of L.P. Se-NPs has been increased from 69 to 100% inhibitory percentage after coating with nano-chitosan at the same MIC of 0.5 ppm. The fungal biomass treated with NCS-Se NPs at the MIC of 0.5 ppm has been reduced to 0.32 ± 0.05 g compared to the treated one with L.P.-Se NPs and CS NPs. The enhanced antifungal efficacy of the nanocomposite NCS-Se NPs causes detected damage to fungal hyphae, introducing a novel, promising, eco-friendly antifungal agent against other plant pathogenic fungi.

## Data Availability

All data generated or analyzed during this study are included in this manuscript.
